# Flow investigation of the stagnation point flow of micropolar viscoelastic fluid with modified Fourier and Fick’s law

**DOI:** 10.1038/s41598-023-36631-1

**Published:** 2023-06-11

**Authors:** Muhammad Naveed Khan, Aamir Abbas Khan, Zhentao Wang, Haifaa F. Alrihieli, Sayed M. Eldin, F. M. Aldosari, Ibrahim E. Elseesy

**Affiliations:** 1 School of Energy and Power Engineering, Jiangsu University, Zhenjiang, 212013 China; 2 Department of Mathematics, University of Sargodha, Sargodha, 40100 Pakistan; 3 Department of Mathematics, Faculty of Science, University of Tabuk, P.O.Box 741, Tabuk, 71491 Saudi Arabia; 4 Faculty of Engineering, Center of Research, Future University in Egypt, New Cairo, 11835 Egypt; 5 Department of Mathematics, College of Science and Humanities in Al-Aflaj, Prince Sattam Bin Abdulaziz University, Al-Kharj, Saudi Arabia; 6 Mechanical Engineering Department, College of Engineering, King Khalid University, Abha, 61421 Saudi Arabia

**Keywords:** Engineering, Materials science, Mathematics and computing

## Abstract

Non-Newtonian fluids are extensively employed in many different industries, such as the processing of plastics, the creation of electrical devices, lubricating flows, and the production of medical supplies. A theoretical analysis is conducted to examine the stagnation point flow of a 2nd-grade micropolar fluid into a porous material in the direction of a stretched surface under the magnetic field effect, which is stimulated by these applications. The stratification boundary conditions are imposed on the surface of the sheet. Generalized Fourier and Fick’s laws with activation energy is also considered to discuss the heat and mass transportation. To obtain the dimensionless version of the flow modeled equations, an appropriate similarity variables are used. These transfer version of equations is solved numerically by the implement of the BVP4C technique on MATLAB. The graphical and numerical results are obtained for various emerging dimensionless parameters and discussed. It is noted that by the more accurate predictions of $\varepsilon$ and M, the velocity sketch is decreased due to occurrence of resistance effect. Further, it is seen that larger estimation of micropolar parameter improves the angular velocity of the fluid.

## Introduction

The collective features of mixed convection and thermal radiation are the abundant consequences in the physiology of human organs such as the heart, liver, and brain. In medicine, science, engineering, and industrial processes, the investigations of mixed convection flow induced by stretching surfaces have a prominent application. These applications have been deliberated by distant investigators. Over a stretched sheet, Khan et al.^1^ investigated the effects of non-linear thermal radiation, viscous dissipation, nonlinear convection, heat sink or source, and thermophoresis on hyperbolic tangent fluid flow with nanoparticles. In nonlinear mixed convection flow of the Newtonian fluid along a heat source or sink, double stratification, and nonlinear thermal radiation beneath the Riga plate, Hayat et al.^2^ examined the mass-heat transmission. Ibrahim and Gizewu^3^ discussed the transportation of heat and mass into the non-Newtonian tangent hyperbolic liquid with nanoparticles of non-linear mixed convection flow with Cattaneo–Christove model with the magnetic effect, activation energy past a non-uniform expandable sheet. Patil et al.^4^ looked at the heat-mass transmission for a water-base fluid flowing in a non-linear mixed convection flow on a vertical cone. Alsaedi et al.^5^ analyzed the heat-mass communication in the Eyring–Powell nanofluid non-linear mixed convection flow with the influence of magnetic effect, Joule heating, and viscous dissipation towards a stretched sheet. By Qasemian et al.^6^, the hydraulic and thermal behaviour of a nanofluid flow inside a tube used in an automated transmission was examined. Fathellahi et al.^7^ looked at how MHD affected the 2D squeezing flow of nanofluid between two evenly spaced sheets. Several studies of the non-linear mixed convection flow of different liquids can be found in Refs.^8–13^.

Due to numerous applications of boundary layer flow in engineering and industrial processes like paper making, plastic sheet and film design, aerodynamic extrusion of plastic and rubber sheets, strengthening and dilution of copper wires, glass fibers, metallic surface cooling in a cooling bath, etc., which caused by a continuous stretched sheet has received significant attention over the past few years. Yurusoy and Pakdemirli^14^ obtained the accurate solutions to the equations regulating the flow of a non-Newtonian fluid towards a stretched sheet. The visco-elastic fluid flow through a permeable media was described by Prasad et al.^15^ as a result of the consequences of reaction rate on the transportation of chemically reactive species towards a stretched sheet. Riaz et al.^16^ addressed the entropy generation impacts and irreversibility comparison flow of Cu-blood nanofluid flow under the applied magnetic field and viscous dissipation impacts through a curved channel. Nadeem et al.^17^ investigated convection and diffusion analysis by mathematical assessment under the viscous dissipation into a noncircular duct. Elgazery and Hassan^18^ investigated the influence of magnetic fields, permeable medium, thermal diffusivity, and variable viscosity for heat-mass transportation into a non-Newtonian liquid on a stretched surface. The heat-mass transmission of stagnation point of a non-Newtonian fluid flow under the effect of heterogeneous and homogeneous chemical processes via an expandable surface was established by Labropulu et al.^19^. Javed et al.^20^ considered the flow of a non-Newtonian liquid with the Powell–Eyring model toward a stretching sheet. The few most current contributions in the stretching sheet may be indicated in the Refs.^21–24^.

The transportation of heat and mass in the flow of non-Newtonian and Newtonian fluids towards a continuously moving sheet are very important in various industries, technological and engineering procedures such as control of the cooling rate, geophysics, crude oil purification, magnetic material processing, a metallic plate that is constantly cooling, discharge of plastic or rubbery sheets, spinning of fibers, glass blowing, continuous casting, taking filament or polymers out of a die, etc. The transmission of heat is a natural phenomenon that occurs due to the variation of temperature among bodies or within the same body. For two centuries, Fourier and Fick’s diffusion laws of diffusion were the prevailing sources to depict the topography of the process of heat-mass communication, as a substitute for visualizing unique mass and thermal diffusion. This is agreeable with the statistic that changing relaxation times for the distribution of velocity should interrupt the temperature along with the concentration sketches. Liu^25^ proved the heat-mass transmission in an MHD flow and created perfect solutions with heat production or absorption and a uniform magnetic field directed at a stretchy sheet. Sanjayanand and Khan^26^ looked at the heat-mass transfer into the laminar flow of a second-grade fluid under the influence of elastic deformation and viscous dissipation through an exponentially stretched sheet. Qasim^27^ scrutinized the collective consequences of heat-mass transmission in the Jaffrey fluid in the existence of a heat sink or source and surface temperature and surface mass convection with a stretching surface. In a MHD, 2-D, constant flow of an incompressible liquid in the existence of a porous material, thermal radiation, and a non-uniform magnetic effect past an extending vertical sheet, Rashidi et al.^28^ considered mass and heat communication. In a rectangular chamber, heated spinning impediments caused convective heat transfer into the viscous fluid flow, which was studied by Nadeem et al.^29^ statistically. Nadeem et al.^30^ analyzed the generalized Fourier and Fick’s law with double diffusion for the axisymmetric stagnation point flow of viscoelastic fluid having nanoparticles on the Riga plate. Ishtiq et al.^31^ analysis of the hybrid nanofluid stagnation point flow towards a stretching/shrinking permeable sheet using the expanded Yavada-Ota and Xue model is based on the effects of MHD. In order to study how heat and mass are transferred into a second-grade fluid containing nanoparticles with the impact of the Cattaneo–Christov double diffusion and buoyancy forces, Nadeem et al.^32^ looked at the variable thermal conductivity and variable viscosity. The importance of heat radiation for the time-dependent two-dimensional flow of third-grade fluid towards a permeable stretching sheet Riga plate was studied by Nadeem et al.^33^. Guedri et al.^34^ discussed the impact of varying thermal conductivity, microrotation, thermal radiation, heat production, heat generation/absorption, and MHD for the flow of third-grade fluid towards an exponentially stretched sheet. Sandeep et al.^35^ provided a comparative study of the heat-mass transmission of Oldroyd-B, Maxwell, and Jaffery fluids having nanoparticles under the effects of suction or injection, Brownian motion, thermophoresis, and transverse magnetic field towards a permeable expanding sheet. Qasim et al.^36^ scrutinized the mass and heat transfer by using Burngiorno model in a thin film of nanofluid towards an unsteadily stretching sheet. For the flow of a hybrid nanofluid in a rotating system, Abdellahi et al.^37^ quantitatively evaluated the mass and heat transfer. The heat and mass communication features of Newtonian and Non-Newtonian liquids were debated in distinct features by investigators for diverse physical parameters in the study^38–50^.

In the present investigation the thermal and solutal transfer phenoneman is considered of the two-dimensional stagnation point flow of micropolar viscoelastic (second grade) fluid with the Cattaneo–Christov heat flux theory towards a stretched sheet. Further we consider to form novelty in the work activation energy, magnetic field, thermophoretic effect, and thermal radiation features. It has not been discussed the such type of issue so far. The numerical solution of the governing PDEs of the flow model after transformation into ODEs is achieved by using the BVP4C Matlab technique. Impacts of various parameters are studied with tables and diagrams across velocity, concentration, micropolar, and temperature distribution.

## Mathematical expression

**Figure 1:**
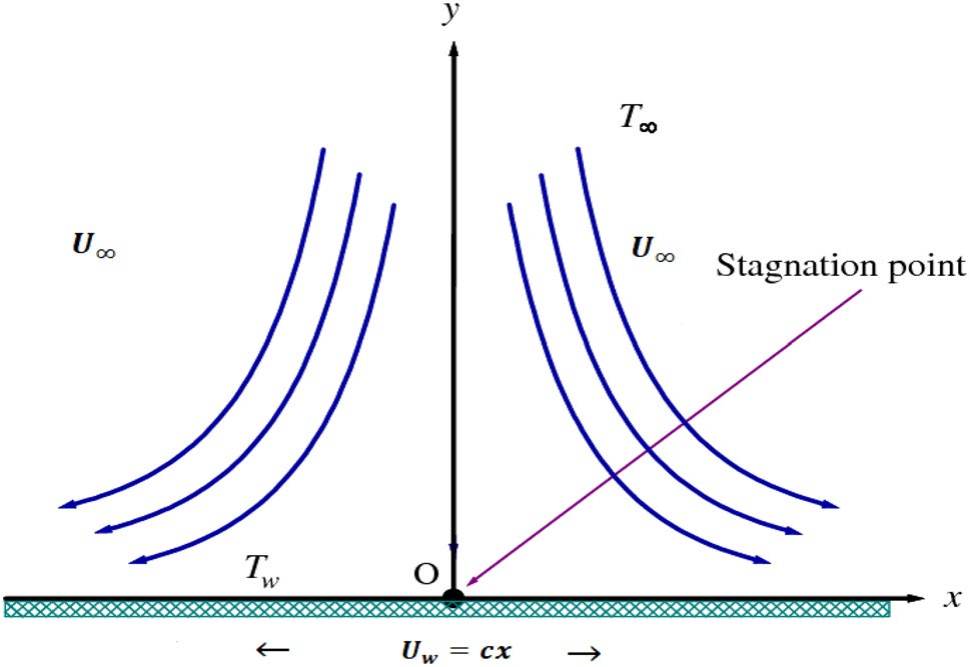
Flow mechanism.

Here we consider a steady, 2D incompressible, stagnation point flow of a micropolar second grade fluid on a stretching surface with modified heat and mass flux. To scrutinize the aspects of mass-heat transportation the thermal radiation and activation energy effects are considered. Further, the thermal and stratification boundary conditions are implemented on the surface of the sheet. The physical model is displayed in the Fig. 1. The sheet is stretching with a velocity of $u=cx$ (here, c is the positive constant). The magnetic effect of strength ${B}_{0}$ is used along the y-direction. The temperature on the sheet is kept up at ${T}_{w}$ and far away from the sheet is ${T}_{\infty }$.

The model of 2nd-grade fluid, the Cauchy stress tensor is defined as^51^,

1 $$T=-PI+\mu {A}_{1}+{\alpha }_{1}{A}_{2}+{\alpha }_{2}{A}_{1}^{2},$$

where $I$ is the identity tensor, $\mu$ is the dynamic viscosity, $P$ is the pressure, ${\alpha }_{i}(i=\text{1,2})$ the material constants, ${A}_{1}$ and ${A}_{2}$ are two Rivlin–Ericksen tensors are

2 $${A}_{1}=\left(grad\, v\right)+{\left(grad\, v\right)}^{T},$$

3 $${A}_{2}=\frac{d{A}_{1}}{dt}+{A}_{1}\left(grad\, v\right)+{\left(grad\, v\right)}^{T}{A}_{1}.$$

Here, $V$ is the velocity and $\frac{d}{dt}$ is the material time derivative. Keep in mind that when the fluid is in equilibrium and at rest locally, the Clausius–Duhem inequality is met, and the Helmboltz free energy is minimal.

4 $$\mu \ge 0, {\alpha }_{1}\ge 0, {\alpha }_{1}+{\alpha }_{2}=0.$$

It should be noticed that the constitutive equation of a fluid in the second grade simplifies to an equation for a viscous fluid when ${\alpha }_{1}={\alpha }_{2}=0$.

The momentum, continuity, and heat equations with some effects are as followed^52,53^.

5 $$\frac{\partial u}{\partial x}+\frac{\partial v}{\partial y}=0,$$

6 $$u\frac{\partial u}{\partial x}+v\frac{\partial u}{\partial y}={U}_{\infty }\frac{d{U}_{\infty }}{dx}+\left(\frac{\mu +k}{\rho }\right)\frac{{\partial }^{2}u}{\partial {z}^{2}}+\frac{k}{\rho }\frac{\partial N}{\partial z}+\frac{1}{\rho }\sigma {B}_{0}^{2}\left({U}_{\infty }-u\right)-\frac{\nu }{{k}_{1}}u+\frac{{\alpha }_{1}}{{c}_{p}\rho }\left[\frac{\partial }{\partial x}\left(u\frac{{\partial }^{2}u}{\partial {y}^{2}}\right)+\frac{\partial u}{\partial y}\frac{{\partial }^{2}v}{\partial {v}^{2}}+v\frac{{\partial }^{3}u}{\partial {y}^{3}}\right],$$

7 $$u\frac{\partial N}{\partial x}+v\frac{\partial N}{\partial y}=\frac{\gamma }{j\rho }\frac{{\partial }^{2}N}{\partial {y}^{2}}-\frac{k}{j\rho }\frac{\partial v}{\partial z}-\frac{2k}{j\rho }N,$$

8 $$u\frac{\partial T}{\partial x}+v\frac{\partial T}{\partial y}+{\lambda }_{E}\left[\left(u\frac{\partial u}{\partial x}+v\frac{\partial u}{\partial y}\right)\frac{\partial T}{\partial x}+\left(v\frac{\partial v}{\partial y}+u\frac{\partial v}{\partial y}\right)\frac{\partial T}{\partial y}+2uv\frac{{\partial }^{2}T}{\partial x\partial y}+{u}^{2}\frac{{\partial }^{2}T}{\partial {x}^{2}}+{v}^{2}\frac{\partial v}{\partial x}\right]=\alpha \frac{{\partial }^{2}T}{\partial {y}^{2}}+\frac{{\alpha }_{1}}{\rho {c}_{p}}\left[\frac{\partial u}{\partial y}\left[\frac{\partial }{\partial y}\left(u\frac{\partial u}{\partial x}+v\frac{\partial u}{\partial y}\right)\right]\right]+\left(\frac{\mu +k}{\rho {c}_{p}}\right){\left(\frac{\partial u}{\partial y}\right)}^{2}-\frac{1}{\rho {c}_{p}}\frac{\partial {q}_{r}}{\partial y},$$

9 $$u\frac{\partial C}{\partial x}+v\frac{\partial C}{\partial y}+{\lambda }_{c}\left[\left(u\frac{\partial u}{\partial x}+v\frac{\partial u}{\partial y}\right)\frac{\partial C}{\partial x}+\left(u\frac{\partial v}{\partial y}+v\frac{\partial v}{\partial y}\right)\frac{\partial C}{\partial y}+2uv\frac{{\partial }^{2}C}{\partial x\partial y}+{u}^{2}\frac{{\partial }^{2}C}{\partial {x}^{2}}+{v}^{2}\frac{{\partial }^{2}C}{\partial {y}^{2}}\right]={D}_{B}\frac{{\partial }^{2}C}{\partial {y}^{2}}-{{K}_{r}}^{2}{\left(\frac{T}{{T}_{\infty }}\right)}^{m}\text{exp}\left(\left(\frac{-{E}_{a}}{\kappa T}\right)\right)\left(C-{C}_{\infty }\right)-\frac{\partial }{\partial y}\left({V}_{T}C\right).$$

The following boundary criteria apply in each case:

$$u={U}_{w}=cx, v=0, T={T}_{0}+{\in }_{1}x, C={C}_{w}={C}_{0}+{\in }_{2}x, N=-n\frac{\partial u}{\partial y} \text{ at }y\to 0,$$

10 $$u\to {U}_{\infty } , T\to {T}_{\infty }={T}_{0}+{\in }_{3}x, C\to {C}_{\infty }={C}_{0}+{\in }_{4}x, N\to 0 \, {\text{at}} \, y\to \infty .$$

The similarity transformations are as followed,

11 $$u=cx{f}^{^{\prime}}, v=-\sqrt{c\nu }f , N=cxg, \theta =\frac{T-{T}_{\infty }}{{T}_{w}-{T}_{\infty }}, \phi =\frac{C-{C}_{\infty }}{{C}_{w}-{C}_{\infty }}, \eta =\sqrt{\frac{c}{\nu }}y.$$

Here, $\left(u,v\right)$, are the components of velocity corresponding to $\left(x,y\right). \text{The symbols }{U}_{e}$, $\mu$, $k$, $\rho$, $N$, ${c}_{p}$, ${\alpha }_{1}$, $\sigma$, ${B}_{0}$, $g$, ${\lambda }_{1}$, $T$, ${T}_{\infty }$, ${\lambda }_{2}$, ${\lambda }_{3}$, $C$, ${C}_{\infty }$, ${\lambda }_{4}$, $\nu$, ${k}_{1}$, $\gamma$, $j$, ${\lambda }_{E}$, $\alpha$, ${Q}_{0}$, ${q}_{r}$, ${\lambda }_{c}$, ${D}_{B}$, ${k}_{r}$, $m$, ${E}_{a}$, ${U}_{m}$, ${T}_{0}$, ${\epsilon }_{1}$, ${\epsilon }_{2}$, ${\epsilon }_{3}$, ${\epsilon }_{4}$, ${C}_{w}$, ${C}_{0}$ and n are represented the free stream velocity, dynamic viscosity, vortex viscosity, density of the fluid, heat capacity, second grade fluid coefficient, electrical conductivity, magnetic field strength, gravity force, linear expansion coefficient, temperature, free stream temperature, non-linear mass expansion, linear mass expansion coefficient, fluid concentration, free stream concentration, non-linear mass expansion coefficient, kinematic viscosity, porous medium permeability, spin gradient, micro-inertia density, relaxation of heat flux, thermal conductivity, coefficient of heat sink/source4, thermal radiation coefficient, the relaxation of mass flux, Brownian motion coefficient, reaction rate coefficient, constant of fitted rate, coefficient of activation energy, wall velocity along x-axis, concentration, reference temperature, positive constants, concentration of fluid at wall, reference concentration and gyration parameter respectively.

The flow expressions in the non-dimensional form become,

12 $$\left(1+K\right){f}^{\prime\prime\prime}+K{g}^{{{\prime}}}-{{f}^{{{\prime}}}}^{2}+f{f}^{\prime\prime}-{M}^{2}\left(\in -{f}^{{{\prime}}}\right)+{\in }^{2}+\beta \left[2{f}^{{{\prime}}}{f}^{\prime\prime\prime}-{{f}^{\prime\prime}}^{2}-f{f}^{\left(iv\right)}\right]-\varepsilon {f}^{{{\prime}}}=0,$$

13 $$\left(1+\frac{K}{2}\right){g}^{\prime\prime}+f{g}^{\prime\prime}-{f}^{{{\prime}}}g-K\left(2g+{f}^{\prime\prime}\right)=0,$$

14 $${P}{r}f{\theta }^{{{\prime}}}-{P}{r}\left(\theta +S\right){f}^{{{\prime}}}-{{P}{r}\delta }_{e}\left[f{f}^{{{\prime}}}{\theta }^{{{\prime}}}+{\theta }^{\prime\prime }{f}^{2}+\left(\theta +S\right)\left({{f}^{{{\prime}}}}^{2}-f{f}^{\prime\prime}\right)\right]+{P}{r}\beta {f}^{\prime\prime}\left[{f}^{{{\prime}}}{f}^{\prime\prime}-f{f}^{\prime\prime}\right]+\left(1+K\right){P}{r}{{f}^{{{\prime}}}}^{2}+{\left[1+R\left({\left(1+\left({\theta }_{w}-1\right)\theta \right)}^{3}\right){\theta }^{{{\prime}}}\right]}^{{{\prime}}}=0,$$

15 $${\phi }^{\prime\prime}-{Sc}\left(\phi +{S}^{*}\right){f}^{\prime}+{Sc}{f}{\phi }^{{{\prime}}}-{Sc}{\delta }_{c}\left[f{f}^{{{\prime}}}{\phi }^{{{\prime}}}+{\phi }^{\prime\prime }{f}^{2}+\left(\phi +{S}^{*}\right)\left({{f}^{{{\prime}}}}^{2}-f{f}^{\prime\prime}\right)\right]+{Sc}{\epsilon }_{5}{\left(1+{\epsilon }_{6}\right)}^{m}{e}^{-\frac{E}{\left(1+{\epsilon }_{6}\theta \right)}}\phi -{Sc}\tau \left({\theta }^{{{\prime}}}{\phi }^{{{\prime}}}+{\theta }^{\prime\prime}\phi \right)=0.$$

The comparable boundary conditions are as follows:

$$f=0, {f}^{^{\prime}}=0, \theta =1-S , \phi =1-{S}^{*}, g=-n{f}^{\prime\prime}\left(0\right)\text{ as }\eta \to 0,$$

16 $${f}^{{{\prime}}}\to 1 , \theta \to 0, \phi \to 0, g\to 0\text{ at }\eta \to \infty ,$$

where, $K=\frac{k}{\mu }$ (Micropolar fluid parameter), $M=\frac{{\sigma }_{0}{B}_{0}^{2}}{\rho c}$ (Magnetic effect parameter), $\varepsilon =\frac{a}{c}$ (Velocity ratio parameter), $\beta =\frac{{\alpha }_{1}c}{\nu }$ (Second grade fluid parameter), $\in =\frac{\nu }{{k}_{0}c}$ (Porous medium parameter), ${L}_{e}=\frac{k}{\rho {c}_{p}{D}_{B}}$ (Lewis number), ${S}_{c}=\frac{\nu }{{D}_{B}}$ (Simdth number), ${\delta }_{c}={\lambda }_{c}c$ (Concentration relaxation time parameter), ${\in }_{5}=\frac{{k}_{r}^{2}}{c}$ (Parameter of chemical reaction), ${\in }_{6}=\frac{{T}_{w}-{T}_{\infty }}{{T}_{\infty }}$ (Parameter of temperature difference), $E=\frac{{E}_{a}}{\kappa {T}_{\infty }}$ (Activation energy parameter), $S=\frac{{\in }_{3}}{{\in }_{1}}$ (Thermal stratification parameter), ${S}^{*}=\frac{{\in }_{4}}{{\in }_{2}}$ (Concentration stratification parameter), ${P}{r}=\frac{\rho {c}_{p}\nu }{k}$ (Prandtl number), $R=\frac{16{\sigma }^{*}{T}_{\infty }^{3}}{3k{k}^{*}}$ (Radiation parameter), ${\theta }_{w}=\frac{{T}_{w}}{{T}_{\infty }}$ (Temperature ratio parameter), ${\delta }_{e}={\lambda }_{E}c$ (thermal relaxation time parameter).

### Skin friction

From an engineering perspective, skin friction is very important physical quantities, which is stated by,

17 $${C}_{fx}= \frac{{\tau }_{w}}{\frac{1}{2}\rho {{u}_{w}}^{2}},$$

18 $${\tau }_{w}={\left[\frac{{\alpha }_{1}}{\rho }\left(u\frac{{\partial }^{2}u}{\partial {y}^{2}}-2\frac{\partial u}{\partial x}\frac{\partial v}{\partial y}+u\frac{{\partial }^{2}u}{\partial x\partial y}\right)+ \left(\frac{\mu +k}{\rho }\right)\frac{\partial u}{\partial y}+k{N}_{1}\right]}_{|y=0}.$$

In the dimensionless form,

19 $${{C}_{fx}}_{|\eta =0}=\left[\beta \left(3{f}^{{{\prime}}}\left(0\right){f}^{\prime\prime}\left(0\right)-{ff}^{\prime\prime{^{\prime}}}\left(0\right)\right)+(1+K)f{^{\prime}}{^{\prime}}(0)\right]{\left(\frac{{R}_{ex}}{2}\right)}^{-\frac{1}{2}},$$

where ${R}_{ex}=\frac{{xu}_{w}}{\nu }$ is the Reynolds number.

## Methodology

**Figure 2:**
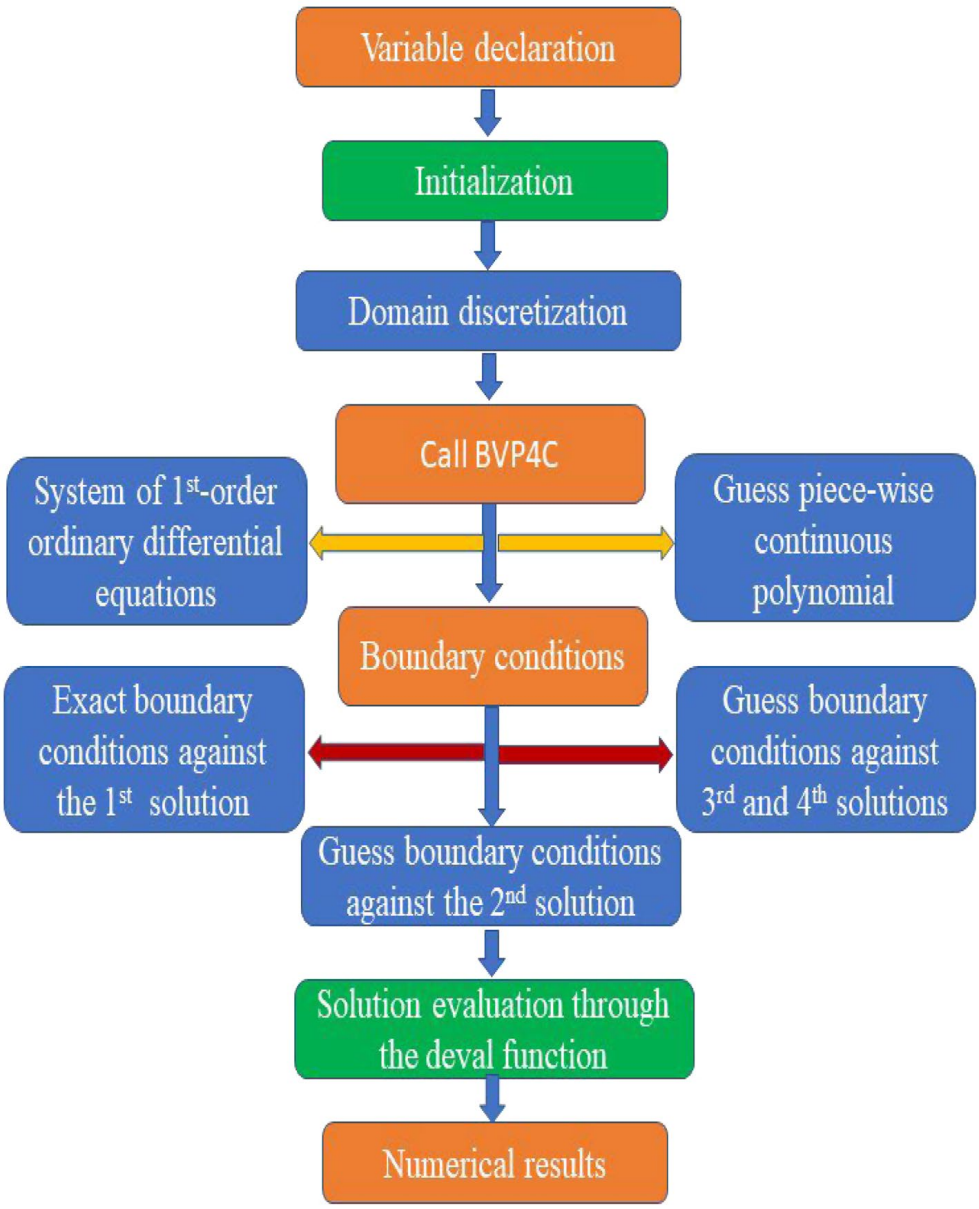
Numerical scheme.

In this section, the two-dimensional viscoelastic fluid flow with modified heat and mass flux toward a stretching sheet is inspected numerically by using the BVP4C MATLAB approach. To used BVP4C first we convert the nonlinear ordinary differential equations into form system of first order differential equations. For this conversion we consider the momentum, micropolar, temperature, and concentration Eqs. (12)–(15) with boundary equation conditions (16) and convert them into the new variables. Moreover, numerical scheme is given in Fig. 2. In this instance, the new variables are introduced as follows:

23 $$\begin{array}{*{20}l} {f = y_{1} ,f^{\prime\prime\prime} = y_{4} , f^{\prime} = y_{2} ,f^{\prime\prime} = y_{3} ,f^{{\prime\prime\prime\prime}} = yy_{1} } \hfill \\ {g = y_{5} , g^{\prime} = y_{6} ,g^{\prime\prime} = yy_{2} } \hfill \\ {\theta = y_{7} , \theta^{\prime} = y_{8} ,\theta^{\prime\prime} = (y_{8}){\prime} } \hfill \\ {\phi = y_{9} , \phi^{\prime} = y_{10} ,\phi^{\prime\prime} = yy_{4} } \hfill \\ \end{array} ,$$

24 $$y{y}_{1}=\frac{1}{\beta }\left\{K{y}_{6}-\beta \left({y}_{3}^{2}-2{y}_{2}{y}_{4}\right)-\left(1+K\right){y}_{4}-{y}_{2}^{2}+{y}_{1}{y}_{3}-{M}^{2}\left(\in -{y}_{2}\right)+{\in }^{2}-\varepsilon {y}_{2}\right\},$$

25 $$y{y}_{2}=\frac{1}{\left(1+\frac{K}{2}{y}_{1}\right)}\left\{{y}_{1}{y}_{6}+K\left(2{y}_{5}+{y}_{3}\right)\right\},$$

26 $${\left({y}_{8}\right)}^{^{\prime}}+{P}{r}{\left(\left(1+R\left({\left(1+\left({\theta }_{w}-1\right)\theta \right)}^{3}\right)\right){y}_{8}\right)}^{^{\prime}}=\frac{1}{\left(1+{P}{r}{S}_{c}{y}_{1}^{2}\right)}\left\{{P}{r}{\delta }_{e}\left[{y}_{1}{y}_{2}{y}_{8}+\left({y}_{7}+S\right)\left({y}_{2}^{2}-y{y}_{3}\right)\right]-{P}{r}{y}_{1}{y}_{8}\right\}-{P}{r}\beta {y}_{3}\left({y}_{2}{y}_{3}-{y}_{1}{y}_{3}\right)-\left(1+K\right){P}{r}{y}_{2}^{2},$$

27 $$y{y}_{4}=\frac{1}{\left(1+{S}{c}{y}_{1}^{2}-{P}{r}{\delta }_{c}{y}_{1}^{2}\right)}\left\{{S}{c}{y}_{1}{y}_{2}{y}_{10}+{P}{r}{S}{c}\left({y}_{1}{y}_{2}{y}_{10}+\left({y}_{9}+{S}^{*}\right)\left({y}_{2}^{2}-{y}_{1}{y}_{3}\right)-{S}{c}{\epsilon }_{5}{\left(1+{\epsilon }_{6}\right)}^{m}{e}^{-\frac{E}{\left(1+{\epsilon }_{6}{y}_{7}\right)}{y}_{9}}+{S}{c}\tau \left({y}_{8}{y}_{10}+y{y}_{3}{y}_{9}\right)\right)\right\}.$$

## Numerical results

In this section the details analysis of numerical data is pretested in the Table 1 for several physical parameters, such as $K, M, {\beta }_{t},$ and $\varepsilon$ along the skin friction. According to Table 1, it is noted that greater estimation of $K$ and $M$ leads to higher $f{{\prime}}(0)$ values. Table 1 illustrates the impact of ${\beta }_{t}$ on the $f{{\prime}}(0)$. It is observed that greater values of ${\beta }_{t}$ and $\varepsilon$ leads to higher estimates of skin friction $f{{\prime}}(0)$. The comparison between the current findings of the second-grade fluid parameter influence on skin friction and Rafiq et al.^54^ is shown in Table 2. The results of the present study shows great harmony with previous data. This comparison gives us confidence in the results.

**Table 1:** The estimations of the skin friction coefficient for the stronger estimations of $K, M, {\beta }_{t}$, and $\varepsilon$.

$K$	$M$	${\beta }_{t}$	$\varepsilon$	${C}_{{f}_{x}}$
$0.1$				1.4423
$0.2$				1.7560
$0.3$				1.6374
	$0.1$			1.5337
	$0.2$			1.7613
	$0.3$			2.0920
		$0.1$		1.8363
		$0.2$		1.8774
		$0.3$		1.9374
			$0.1$	15.7372
			$0.2$	16.3725
			$0.3$	17.0864

**Table 2:** Numerical results of skin friction compared with present results.

$\beta$	Present results	Rafiq et al.^54^
	${f}^{\prime\prime}(0)$	${f}^{\prime\prime}(0)$
0.0	− 2.2537	− 2.24676880475314
0.2	− 4.1226	− 4.09820479923631
0.5	− 6.9593	− 6.93689203353080
0.7	− 9.4330	− 9.40436723010559
1.0	− 13.8760	− 13.8596936496045
2.0	− 59.8754	− 59.2327101256866

## Graphical analysis

The following illustrations are created in order to examine variation of various parameters on the linear and angular velocity, temperature, and concentration profiles. Different estimations are given for the $\beta , M, \varepsilon , K, {\delta }_{e}, {P}{r}, R, {\theta }_{w}, S, {S}{c}, E$, and ${S}^{*}$ to see the effects of them on heat transfer and fluid flow.

### Linear and angular velocity profile analysis

**Figure 3:**
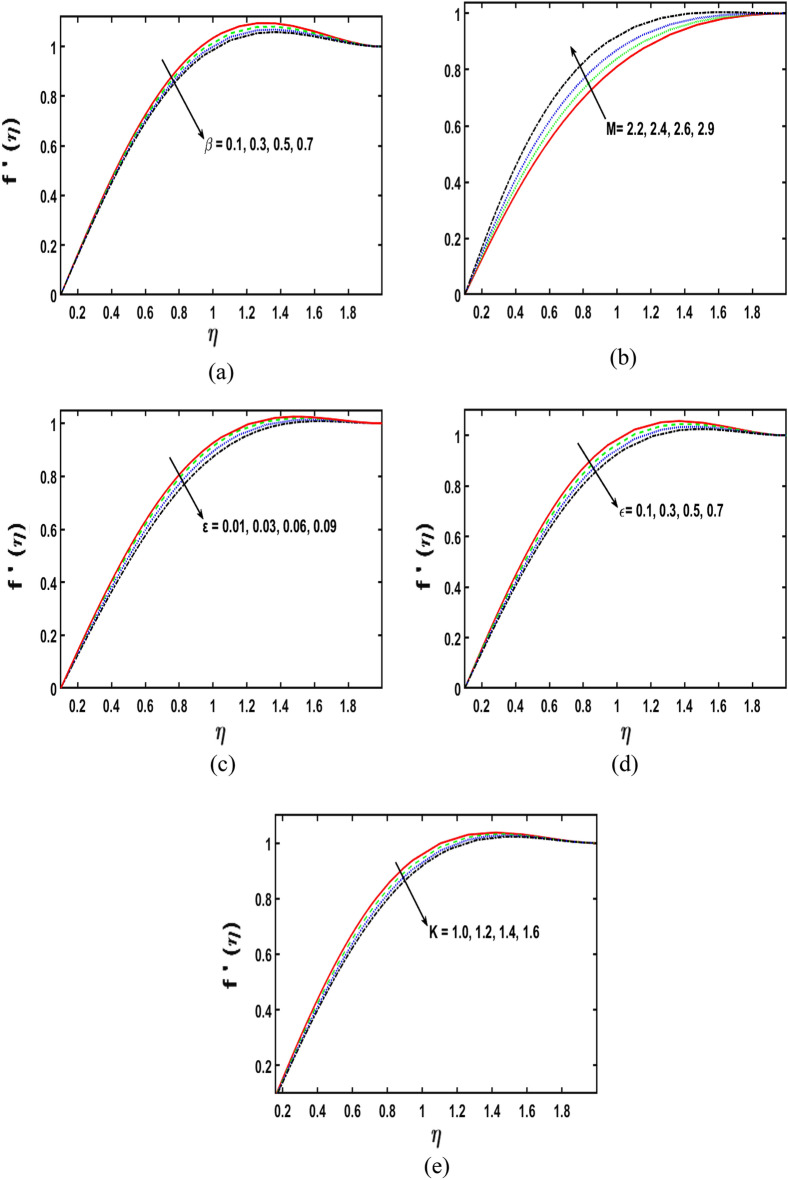
(**a–e**) Deviation in $\beta , M, \varepsilon , \epsilon$, and $K$ along the velocity profiles.

**Figure 4:**
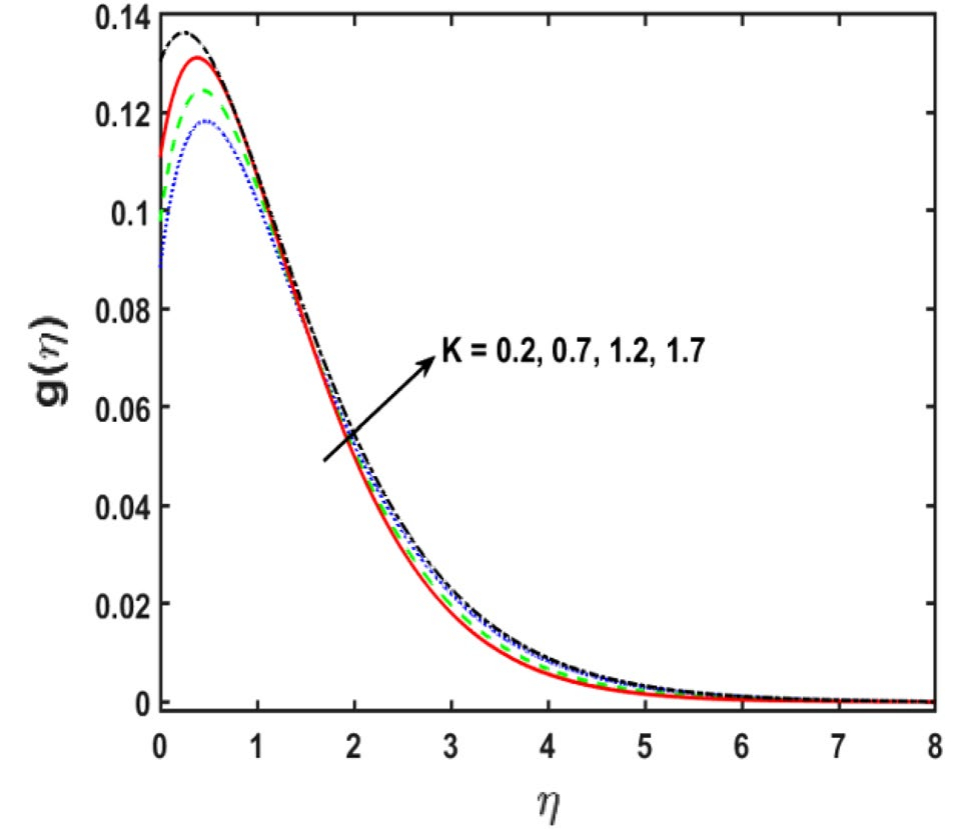
Deviation in $K$ for micropolar profile.

The effect of the viscoelastic fluid parameter $(\beta )$ on the velocity of the second-grade micropolar fluid in the boundary layer is revealed in Fig. 3a. The velocity of fluid rises when the viscoelastic fluid parameter is given greater values. Physically, by enhancing the estimations parameter of the viscoelastic fluid, the viscosity of fluid diminishes, therefore, that creates the momentum boundary layer dense. The velocity sketch effect on the magnetic field parameter (M) is perceived in Fig. 3b. It is noticed that the fluid velocity declines as the magnetic field parameter $\left(M\right)$ gets stronger estimated. Physically, upsurge with the magnetic field parameter $\left(M\right)$, upshots in a dominant decrease inside the corresponding velocity. Physically, the magnetic effect makes a retarding body force that is renowned as Lorentz force that creates more resistance to the velocity of the fluid. Moreover, this force produces more resistance to the mass transportation phenomenon. Figure 3c illustrates the influence of the parameter of stagnation point flow $\left(\varepsilon \right)$ on the fluid velocity draft. By the stronger values of $\left(\varepsilon \right)$, the velocity sketch decreases. Physically, the velocity distribution and the corresponding boundary layer thickness are boosted, for stronger estimations of $\varepsilon$, therefore, the velocity sketch reduces. Figure 3d displays the performance of porous medium parameter $\left(\epsilon \right)$ on the velocity sketch. It is distinguished that the velocity profile reduces for aggregating the variations of porous medium parameter $\left(\epsilon \right)$. Figure 3e illustrates the impact of the micropolar fluid parameter $(K)$ on the velocity sketch. It is perceived that for $K$, the velocity sketch boosts as the upsurge in the variations of the estimations of $K$. Therefore, It is obvious that the boundary layer thickness augmented for $K$. The relationship between the angular velocity and the micropolar fluid parameter is shown in the Fig. 4. The figure illustrates that when the estimates of the micropolar fluid parameter K improves, the angular velocity also increases.

### Temperature profile analysis

**Figure 5:**
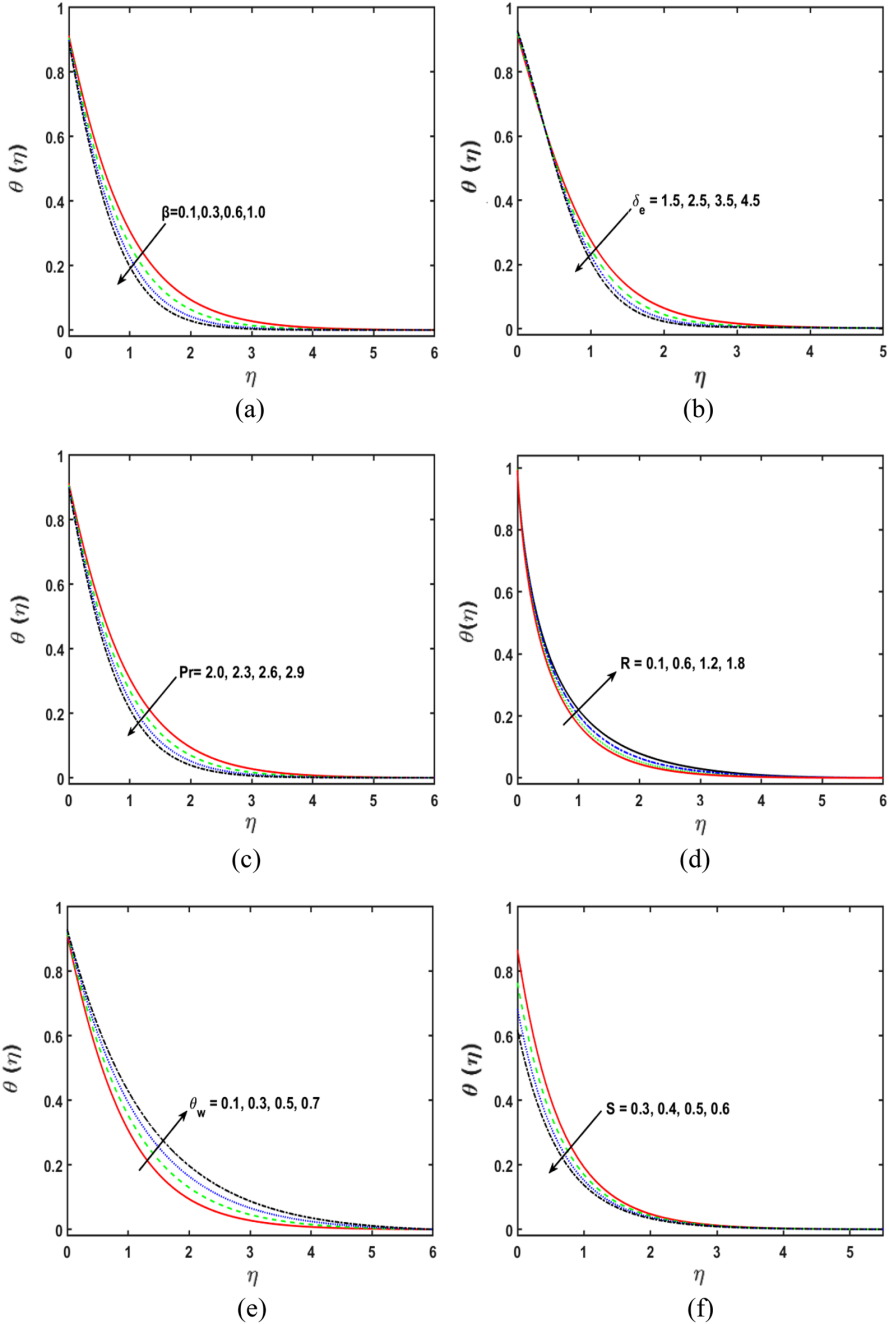
(**a–g**) Deviation in $\beta , {\delta }_{e}, {P}{r}, R, {\theta }_{w}, and \, S$ along temperature profile.

The consequence of viscoelastic fluid parameter $\beta$ on the temperature sketch in Fig. 5a. It is found that with an upsurge in the second-grade fluid parameter, the temperature profile grows, consequently, the corresponding thermal boundary layer rises. From Fig. 5b, it is noted that the temperature field declines for the stronger variations of the thermal relaxation parameter ${\delta }_{e}$. Physically, the parameter of the time relaxation gives the corresponding condition for temperature exchange and decays the boundary layer thickness. Consequently, the liquid viscosity will be a little increased. The behavior of $\theta (\eta )$ for the improving estimations of ${P}{r}$ is shown in the Fig. 5c. The thick dispersion frequency to the thermal dispersion frequency is therefore defined as the ${P}{r}$. The aftereffects of the non-linear thermal radiation parameter $(R)$ on the $\theta (\eta )$ are observed in Fig. 5d. It is displayed that the $\theta (\eta )$ enhances owing to the improving estimations of thermal radiation parameter. The increasing values of the thermal radiation parameter are what determine the thickness of the thermal boundary layer. Figure 5e depicts how the temperature ratio parameter affects the temperature sketch. It is depicted that the temperature profile increases due to the stringer estimations of temperature ratio parameter ${\theta }_{w}$. Figure 5f displays the variation of temperature sketch to see the effect of thermal stratification parameter $S$ on temperature profile. The temperature sketch decreases for greater estimates of the thermal stratification parameter S. Physically, when the estimates of S are improved, the thermal boundary layer thickness decreases.

### Concentration profile analysis

**Figure 6:**
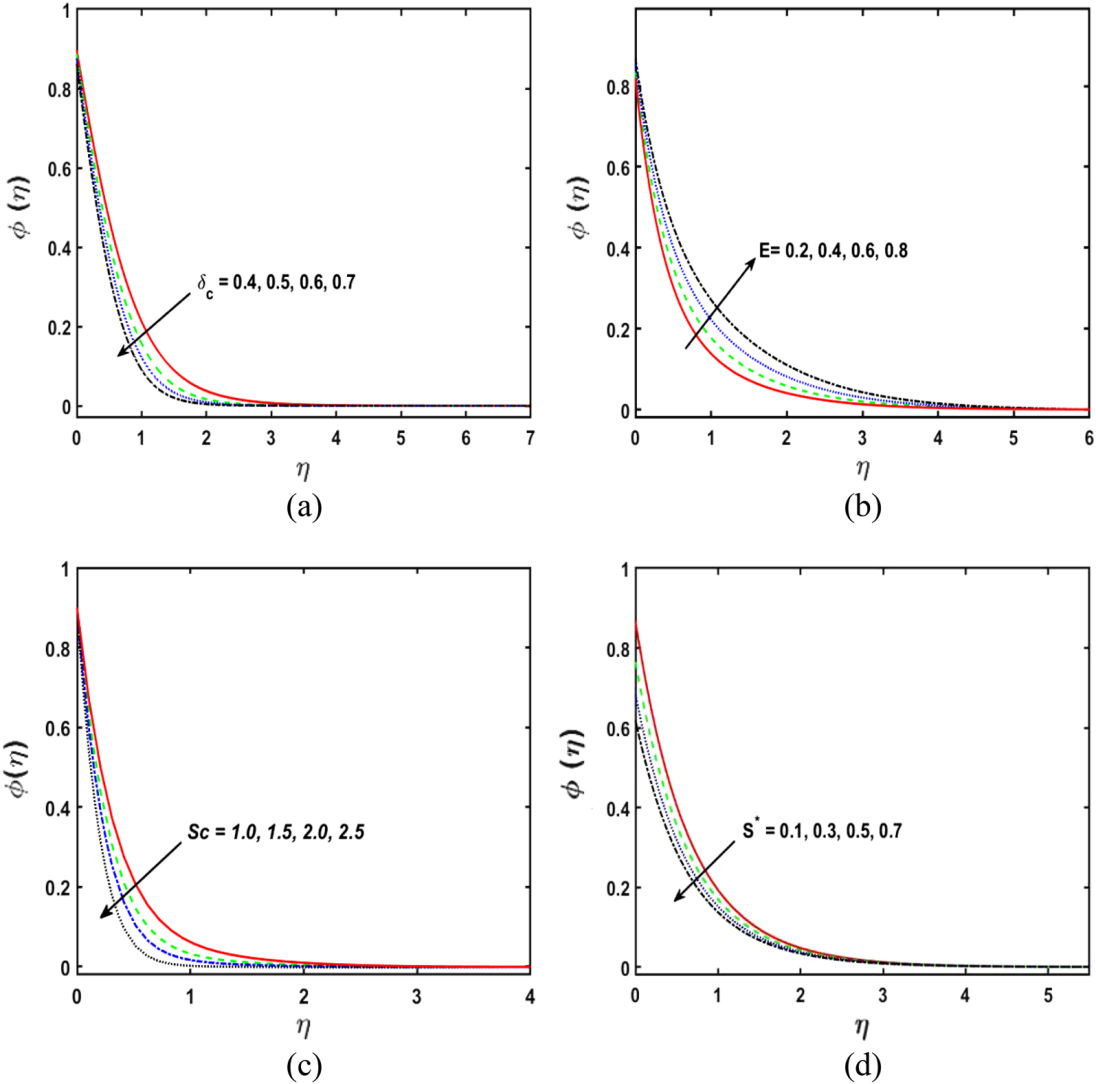
(**a,d**) Deviation in ${\delta }_{c}$, $E$, ${S}{c}$, and ${S}^{*}$ vs concentration profile.

Figure 6a demonstrates the upshot of the concentration relaxation parameter ${\delta }_{c}$ on the $\phi \left(\eta \right)$ draft. It is clear that by enhancing the concentration relaxation parameter ${\delta }_{c}$, the concentration profile diminishes. Physically, by boosting the estimations of ${\delta }_{c}$, the concentration boundary layer acquires thinner. Figure 6b describes the influence of the $E$ (activation energy parameter) on the concentration sketch. By improving the parameter of the activation energy parameter, the increment is observed in the concentration sketch. Physically, the Arrhenius function diminishes if the activation energy parameter $E$ upsurge. Figure 6c depicts the impact of Schmidt number ${S}{c}$ on the concentration outline. Through raising the estimates of Schmidt number ${S}{c}$, a decrement is seen in the concentration sketch. Since the Schmidt number is defined as the proportion of momentum to mass diffusivity. Figure 6d explains the deviation of concentration sketch under the impact of concentration stratification parameter ${S}^{*}$. It is seen that, by growing the parameter of the concentration stratification parameter ${S}^{*}$ the concentration sketch reduces.

## Concluding remarks

In the present research article, the heat and mass transport analysis of 2D stagnation point flow of micropolar second-order fluid past a stretchable sheet with Cattaneo–Cristove heat flux theory and stratification effect are examined numerically. Some useful outcomes of this work are given as,

- The enhancement in the velocity sketch is noticed for the stronger estimates of the 2nd-grade fluid parameter ($\beta$).
- With stronger M and $\varepsilon$ estimates, declines in the velocity sketch.
- The velocity profile is improved by the increasing the estimation of K.
- The angular velocity of the fluid upsurges due to the stronger values of $K$.
- The stronger estimations of $\beta$ and ${\delta }_{e}$ leads to the decay of the temperature sketch.
- The temperature profile diminishes for ${P}{r}$ and improves for stronger estimation of $R$.
- As higher values of thermal stratification parameter the temperature profile increases.
- The skin friction coefficient shows an increasing behavior for improving values of $K, \beta , M$, and $\epsilon$.
- For larger values of ${S}^{*}$ declines the concentration profile.

## Data Availability

The data that support the findings of this study are available from the corresponding author upon reasonable request.
